# Nutrient‐dependent allometric plasticity in a male‐diphenic mite

**DOI:** 10.1002/ece3.9145

**Published:** 2022-08-01

**Authors:** Flor T. Rhebergen, Kathryn A. Stewart, Isabel M. Smallegange

**Affiliations:** ^1^ Institute for Biodiversity and Ecosystem Dynamics University of Amsterdam Amsterdam The Netherlands; ^2^ Institute of Environmental Sciences Leiden University Leiden The Netherlands; ^3^ School of Natural and Environmental Sciences Newcastle University Newcastle upon Tyne UK

**Keywords:** acarid mites, alternative phenotypes, alternative reproductive tactics, developmental plasticity, developmental threshold, somatic state

## Abstract

Male secondary sexual traits often scale allometrically with body size. These allometries can be variable within species and may shift depending on environmental conditions, such as food quality. Such allometric plasticity has been hypothesized to initiate local adaptation and evolutionary diversification of scaling relationships, but is under‐recorded, and its eco‐evolutionary effects are not well understood. Here, we tested for allometric plasticity in the bulb mite (*Rhizoglyphus robini*), in which large males tend to develop as armed adult fighters with thickened third legs, while small males become adult scramblers without thickened legs. We first examined the ontogenetic timing for size‐ and growth‐dependent male morph determination, using experimentally amplified fluctuations in growth rate throughout juvenile development. Having established that somatic growth and body size determine male morph expression immediately before metamorphosis, we examined whether the relationship between adult male morph and size at metamorphosis shifts with food quality. We found that the threshold body size for male morph expression shifts toward lower values with deteriorating food quality, confirming food‐dependent allometric plasticity. Such allometric plasticity may allow populations to track prevailing nutritional conditions, potentially facilitating rapid evolution of allometric scaling relationships.

## Graphical abstract

The threshold body size for male morph expression in bulb mites Rhizoglyphus robini is food‐dependent. Such allometric plasticity can facilitate rapid evolution of allometric scaling relationships.

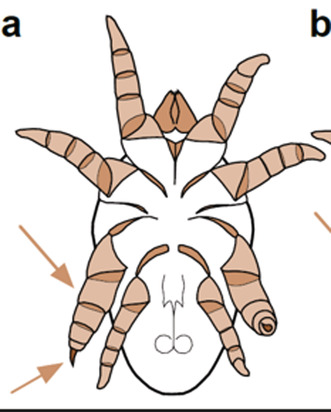

## 1 INTRODUCTION

Male secondary sexual traits are often conspicuously sensitive to variation in juvenile food quality (Emlen & Nijhout, 2000; Kodric‐Brown et al., 2006; Lavine et al., 2015). This nutrition‐sensitive plasticity generally manifests itself in hyper‐allometric scaling of sexual traits with body size (Kawano, 2000; McCullough et al., 2015; Simmons & Tomkins, 1996) or even in discrete alternative reproductive phenotypes (Emlen et al., 2005; Smallegange, 2011). As a consequence, large individuals in a population can have enormous secondary sexual traits, while small individuals can be inconspicuously endowed. Scaling relationships can vary across time and space within species (Miller & Emlen, 2010). Such variation is sometimes due to genetic variation; different populations can differ in genetic background due to drift or local adaptation, resulting in variation in how body size relates to trait size (Buzatto et al., 2012). It is also possible that the scaling relationship itself exhibits plasticity in response to local environmental effects, a phenomenon that has been dubbed “allometric plasticity” (Casasa & Moczek, 2019;Emlen, 1997; Moczek, 2002). Allometric plasticity can occur in response to food variation (Emlen, 1997; Moczek, 2002). In such cases, variation in nutrition not only causes variation in body size and therefore in sexual traits, but also variation in *how* organisms are sensitive to the body size cue to develop sexual traits.

Food‐dependent allometric plasticity can have a significant role in driving ecological and evolutionary patterns in sexual traits because it can create a much larger developmental repertoire than canalized allometric scaling does (Crispo, 2008; Hendry, 2015; West‐Eberhard, 2003). Ecologically, allometric plasticity causes divergence in scaling relationships among populations in different nutritional environments. Such interpopulation variation is often interpreted as local adaptation (Tomkins & Brown, 2004) or speciation in progress (Kawano, 2002), but may instead reflect undetected allometric plasticity. Allometric plasticity can also obscure scaling relationships of interest within populations when environments are nutritionally heterogeneous on small spatial or temporal scales. Such intrapopulation variation in scaling can conceal condition‐dependence, which is often inferred through static allometric scaling (Bonduriansky, 2007; Eberhard et al., 2018), and leave the impression that the association of sexual trait variation with body size is much weaker than it actually is. Evolutionarily, allometric plasticity is hypothesized to be an important first step in adaptive divergence of scaling relationships in novel (nutritional) environments (Casasa & Moczek, 2019; Rohner & Moczek, 2020). Genetic accommodation of ancestrally plastic scaling relationships can allow rapid adaptive evolution, if allometric plasticity in the novel environment coincides with release of selectable cryptic genetic variation, and/or if the novel environment persists for long enough to allow genetic mutations to accumulate and canalize the induced scaling relationship (Casasa & Moczek, 2019; Moczek et al., 2011; Pfennig et al., 2010; West‐Eberhard, 2003).

Despite the hypothesized ecological and evolutionary importance of food‐dependent allometric plasticity, it has been demonstrated only in a few species with continuous positive (=hyperallometric [Shingleton, 2010]) sexual trait allometries (Emlen, 1997; Knell et al., 1999; Moczek, 2002). In the majority of species with allometrically scaling sexual traits, such allometries have been measured in (non‐)randomly sampled individuals from the natural environment, without information on environmental circumstances during development beyond body size (e.g., Buzatto et al., 2014; Kawano, 2000; McCullough et al., 2015). Therefore, to assess whether food‐dependent allometric plasticity affects the eco‐evolutionary dynamics of sexually selected condition‐dependent traits, it is first and foremost necessary to assess whether such allometric plasticity occurs in other well‐known model systems for the evolution of condition‐dependent sexually selected traits.

**FIGURE 1:**
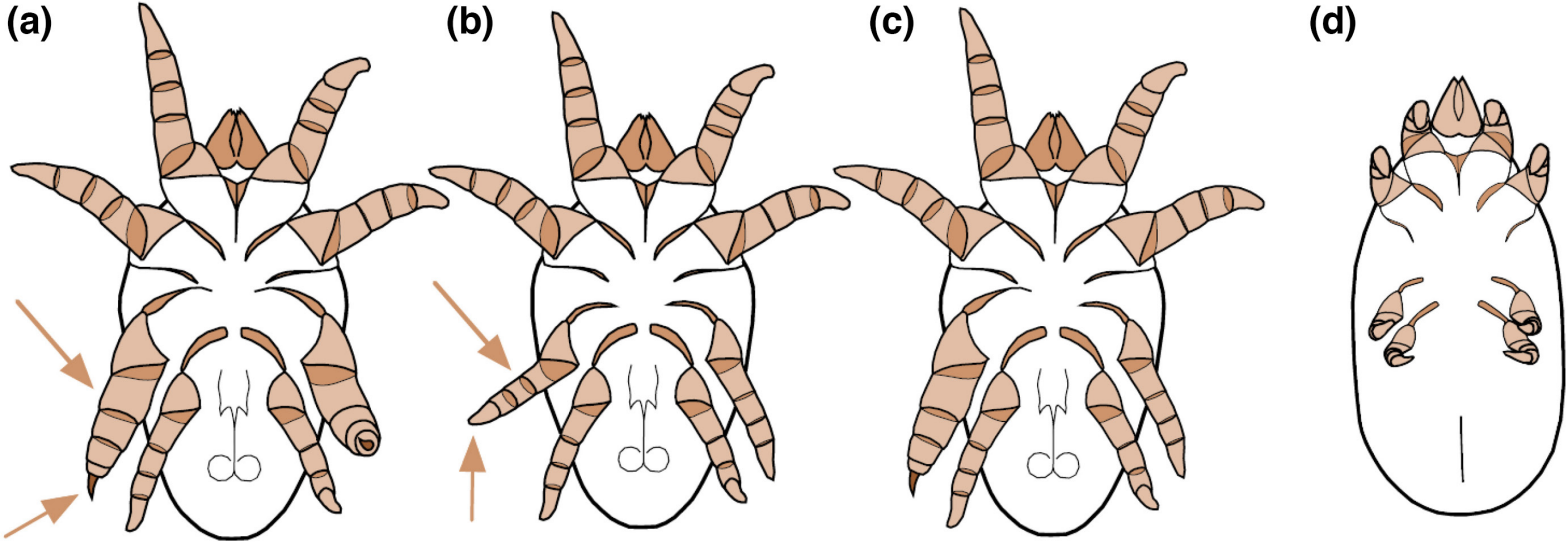
*Rhizoglyphus robini* morphology, ventral view. (a) Adult fighter male. (b) Adult scrambler male. (c) Adult intermorph male. (d) Quiescent tritonymph stage, during which metamorphosis and fighter leg development takes place. Fighters and scramblers differ in the width of the third leg and in the shape of the third tarsal claw (gray arrows)

Here, we tested whether food‐dependent allometric plasticity is present in a species with discrete, condition‐dependent, alternative male morphs: the bulb mite *Rhizoglyphus robini* (Radwan, 1995; Smallegange, 2011). In *R. robini*, large males develop the adult “fighter” morph, which has a proportionally thickened third leg pair with dagger‐like claws that it uses in fights to kill rival males (Radwan et al., 2000; Figure 1a). Small males tend to develop the adult “scrambler” morph, which has a normal, female‐like, third leg pair and a modest motility advantage (Tomkins et al., 2011; Figure 1b). Although it is well established that the alternative morphs in *R. robini* are associated with variation in body size, and thus represent a discontinuous allometric scaling relationship (Leigh & Smallegange, 2014; Smallegange, 2011; Smallegange & Deere, 2014), the correlation is noisy. A large range of overlap in body size exists between juveniles developing as fighters and juveniles developing as scramblers (Smallegange, 2011; Stewart et al., 2018). We hypothesize that these observations are, at least partly, explained by food‐dependent plasticity in how body size scales with male morph expression. Such allometric plasticity, if present, could facilitate the repeatedly observed rapid evolution of how male morph expression relates to body size in *Rhizoglyphus* mites (Casasa & Moczek, 2019; Smallegange & Deere, 2014; Tomkins et al., 2011).

To test the hypothesis that condition‐dependent male morph expression is associated with food‐dependent allometric plasticity, we first identified the sensitive ontogenetic stage at which male body size (or a physiological correlate of it) cues male morph expression. Assuming that growth rate and/or body size explain morph expression best during this sensitive stage, we exposed males to different fluctuating patterns of food availability during ontogeny, creating random variation in growth trajectories from larva to adulthood. We then assessed whether the resulting variation in male morph expression was fully explained by variation in growth rate and/or body size, at different time frames in ontogeny. Having identified the sensitive stage of morph determination, we then tested whether the allometry of adult third leg width and body size at the sensitive stage of morph determination shifts with food quality. We find that adult male morph expression is cued by growth rate and body size immediately before metamorphosis, and that this threshold body size at metamorphosis shifts toward smaller values with deteriorating food quality. Such allometric plasticity in *R. robini* can allow the body size threshold for male morph expression to adaptively track nutritional conditions and resulting shifts in the body size distribution.

## 2 METHODS

### 2.1 Study species and general procedures

*Rhizoglyphus robini* is a cosmopolitan agricultural pest mite on numerous crops and forms dense populations on subterraneous plant structures (Díaz et al., 2000). The populations in this study were derived from *R. robini* samples that were collected from flower bulbs in storage rooms in Noord‐ Holland, The Netherlands, in December 2010. In favorable environments, *R. robini* has four morphological life history stages (larva, protonymph, facultative deutonymph, tritonymph, then adult), interspersed with quiescent phases during which individuals do not move or eat, but undergo incomplete metamorphosis to enter the next stage (Figure 1d).

We kept our *R. robini* populations in an unlit climate cabinet (25°C; >90% humidity), in plastic containers (10 × 10 × 2.5 cm) with a plaster of Paris substrate. Twice a week, we cleaned one‐sixth of the substrate by removing all organic matter and added fresh water and food. We kept half of our populations (four containers) on a diet of baker's yeast and the other half on a diet of oats. Egg‐to‐ egg time averages 11 days on yeast and 13 days on oats when food is ad libitum. Otherwise, mites from both diets are similar; no morphological or behavioral differences have been recorded between our yeast‐fed and oats‐fed populations (F. T. Rhebergen, unpublished data). Populations on the same diet are mixed every 6 months to maintain genetic homogeneity.

In the experiments, individual mites were kept in individual plastic tubes (height 5 cm, diameter 1.5 cm) with a moistened plaster substrate. The tubes were sealed with fine mesh, kept in place by a plastic lid, and placed in an unlit climate cabinet (25°C; >90% humidity) throughout the experiment. We avoided bias due to genetic variation by randomly sampling the parental generation from our stocks and allocating one offspring per parental pair to each experimental treatment. Mites were photographed ventrally and measured to the nearest 0.001 μm using a Zeiss Axiocam 105 color camera mounted on a Zeiss Stemi 200‐C stereomicroscope, and ZEN 2 (Blue edition) software.

### 2.2 Experiment 1: When during ontogeny is male morph expression cued by body size and/or growth?

The mites in this experiment were reared on a diet of oats rather than yeast because development is slightly slower on oats than on yeast, increasing our power to find the critical developmental period in which morph expression is determined. The parental generation was formed by collecting 324 individual *R. robini* larvae from the oats‐fed stock populations. The parental larvae were reared to adulthood on a diet of ad libitum oats in individual tubes. Adult females and males (90.1% fighters, 8.3% scramblers, 1.6% intermorphs, that is, males that were fighter on one side and scrambler on the other [Figure 1c]) were randomly paired to produce the focal generation. Pairs were kept in isolation in separate tubes for 3 days and were fed ad libitum oats. From each parental pair, a single egg was collected and individually isolated. These individuals were reared to adulthood on a variable (*n* = 81) or a constant (*n* = 21) diet of powdered oats to obtain a wide range of growth trajectories. Focal individuals that were reared on a variable diet were transferred daily to a fresh tube that either did or did not contain food. The probability of changing to a different environment (i.e., moving from a food to a nonfood tube, and vice versa) was set to 0.8 (*n* = 27), 0.5 (*n* = 27), or 0.2 (*n* = 27). Each individual was photographed and measured at 24‐h intervals to record growth trajectory. We measured idiosoma width and recorded morph expression upon maturation.

To test whether male morph expression was a response to food uptake and growth at the final moments of the tritonymph stage, versus earlier during juvenile growth, we constructed multiple logistic regression models. For each of six developmental stages, we constructed a generalized linear model (GLM) with a logit link function, with morph expression (fighter or scrambler) as response variable, and growth during that stage and size when entering that stage as explanatory variables. The six developmental stages we distinguished were the first and second half of the larval, protonymph, and tritonymph stages (Table 1). We defined “size when entering the first half of the larva stage” as the length of the egg, “size when entering the second half of the larva, protonymph or tritonymph stage” as the idiosoma width of an individual when it had spent half of its total time in its respective stage, and “size when entering the first half of the protonymph or tritonymph stage” as the idiosoma width of the quiescent larva or protonymph, respectively, “growth during a particular stage” as the difference in idiosoma width when entering a particular stage (early/late larva, early/late protonymph or early/late tritonymph) and when entering the following stage. “Growth during the early larval stage” was not available because we did not have idiosoma measurements at hatching. Therefore, we included idiosoma width at the end of the first half of the larval stage, rather than growth during the first half of the larval stage, as the first explanatory variable for the early‐larva logistic regression model. We assessed the performance of the logistic regression models for the different stages by comparing AICc values (Akaike Information Criterion, corrected for small sample size) and calculating the percentage deviance explained. We tested whether the models performed better than chance by comparing the fitted models to null models using LRT. All analyses were done in R v.3.4.4 (R Core Team, 2018), including packages *stats*, *dplyr* (Wickham et al., 2018) and *ggplot2*(Wickham, 2016).

**TABLE 1:** Logistic regression models with male morph expression (response variable), and size at the beginning of a juvenile stage and growth during that stage (explanatory variables), for each juvenile stage

Juvenile stage	AICc	Null deviance	Residual deviance	Difference in deviance (χ^2^, df = 2)	*p*‐value	% deviance explained
Early larva	36.39	30.885 (31 df)	29.533	1.352	.509	4.4%
Late larva	34.30	30.885 (31 df)	27.439	3.446	.179	11.2%
Early protonymph	30.82	29.569 (28 df^a^)	23.860	5.709	.058	19.3%
Late protonymph	32.97	29.569 (28 df^a^)	26.012	3.557	.169	12.0%
Early tritonymph	32.31	30.885 (31 df)	25.455	5.430	.066	17.6%
*Late tritonymph*	*16.37*	*30.885 (31 df)*	*9.516*	*21.369*	*<.001*	*69.2%*

*Notes*: The difference in deviance between the fitted models and null models without any explanatory variables is χ^2^‐distributed under the null hypothesis, with 2 degrees of freedom.

^a^ Three fighter males passed the protonymph stage very quickly precluding multiple protonymph measurements. These males were removed from protonymph models.

### 2.3 Experiment 2: Is size‐dependent male morph expression subject to allometric plasticity?

The parental generation was formed by individually isolating 200 larvae from the yeast‐fed stock populations, in four experimental blocks of 50 larvae each. These larvae were reared to adulthood on a diet of ad libitum yeast in individual tubes. Per block, 15–24 adult males and females were randomly paired in a plastic tube with ad libitum yeast to produce the focal generation. Three days after pairing, eggs of each parental pair were transferred to a clean individual tube without food. Within 12 hours after hatching, larvae were transferred to experimental tubes. Each parental pair contributed a single larva to each food treatment.

The focal individuals were individually isolated and reared to adulthood on quartered ~23 mm^2^ circular disks of filter paper that were soaked for 20 seconds in 10 μl solution of yeast in water. Individuals were subjected to five food treatments, which represented a 2.5‐fold dilution series (yeast solved in water): 40.0 mg/ml (*n* = 69), 16.0 mg/ml (*n* = 40), 6.40 mg/ml (*n* = 40), 2.56 mg/ml (*n* = 69), and 1.02 mg/ml (*n* = 40). Experimental blocks 1 and 4 contained all treatments, but due to logistical constraints, blocks 2 and 3 only contained the 40 mg/ml and 2.56 mg/ml treatments. Every 2 days, the substrate of each tube was cleaned with a moist brush and the yeast‐soaked disk was replaced with a fresh disk. Because mites feed and survive on filter paper but can never singly consume one disk in 2 days (Smallegange, 2011), this dilution series offers ad libitum food of different quality. In this way, we minimize variation in food chemistry as we keep the food type as similar as possible. After individuals had entered the quiescent tritonymph stage, the disk was removed; adults emerged in an empty tube.

Each individual was photographed and measured in the quiescent tritonymph (Figure 1d) and adult stage (Figure 1a–c). We measured the width of the idiosoma (the white bulbous posterior part of the body) in the quiescent tritonymphs and adults (st. dev. = 3.3 μm; Supplementary Information) and the width of both third leg femurs in the adults (st. dev. = 1.1 μm) and recorded male morph expression.

To investigate the effect of food quality and quiescent tritonymph idiosoma width, QTIW, on the probability of fighter expression, we constructed a logistic regression model with fighter expression as response variable, QTIW, food quality, the interaction between QTIW and food quality and block as fixed explanatory variables and parent ID as random intercept. Four intermorph males were excluded from this analysis. We centered QTIW around the overall mean QTIW value, so that the intercept represented the log‐odds of fighter expression at the average QTIW. We tested significance of explanatory variables using LRT.

## 3 RESULTS

### 3.1 Experiment 1: When during ontogeny is male morph expression cued by body size and/or growth?

**FIGURE 2:**
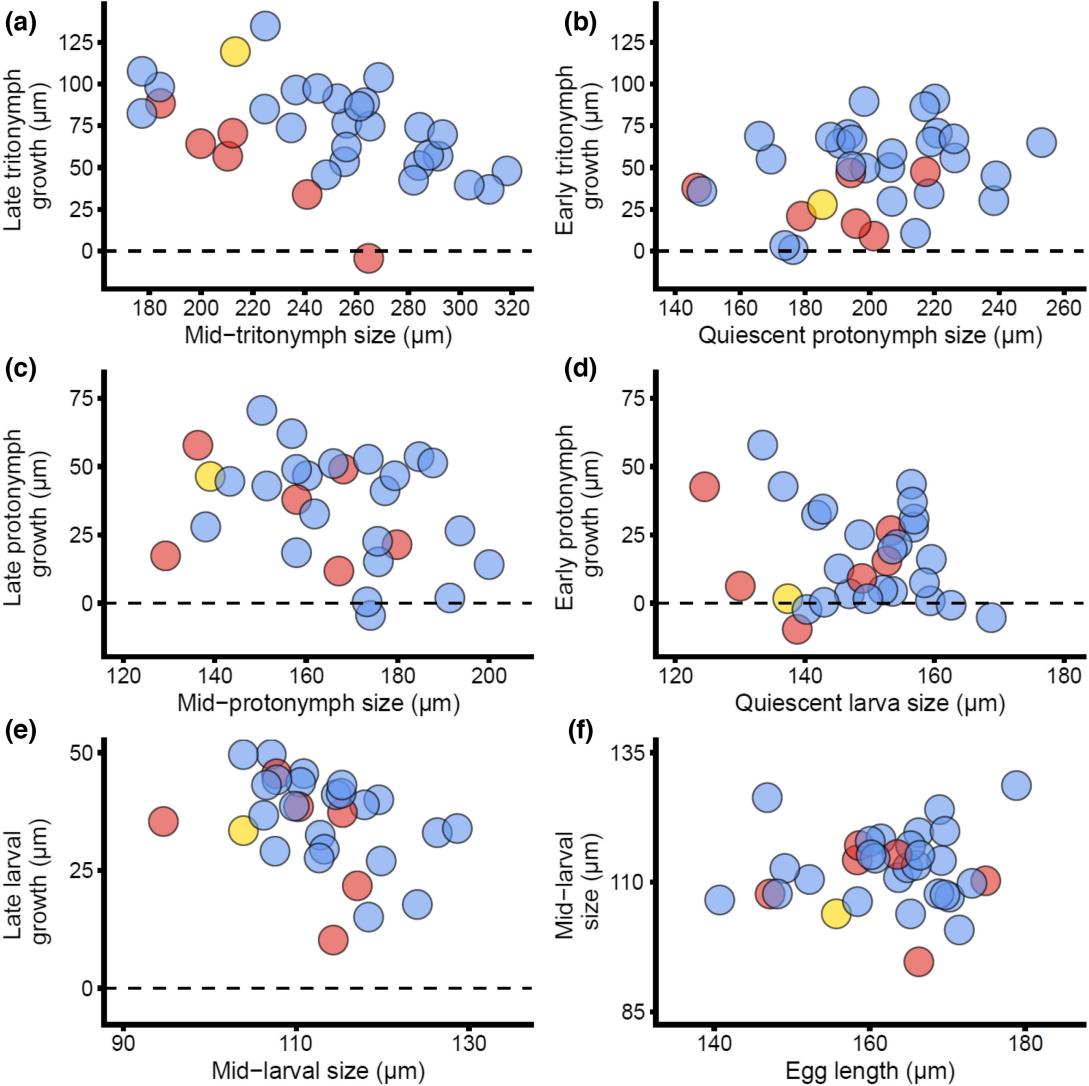
Male morph expression (blue: fighter, red: scrambler, yellow: intermorph) as a response to experimentally induced variation in size at the beginning of a particular juvenile developmental stage, and growth during that stage. (a) Second half of the tritonymph stage. (b) First half of the tritonymph stage. (c) Second half of the protonymph stage. (d) First half of the protonymph stage. (e) Second half of the larval stage. (f) First half of the larval stage

We were able to follow the developmental trajectories of 69 focal individuals from egg to adulthood (67.6% of total isolated eggs). The individuals that we were not able to follow either died during experimentation or were accidentally lost during handling. Of these 69 individuals, 33 were males, 26 of which expressed the fighter morph. Adult morph expression was predicted by idiosoma growth and size in the last half of the tritonymph stage, immediately prior to metamorphosis (Figure 2a) (GLM: 69.2% deviance explained, χ^2^ = 21.37, df = 2, *p* < .001; Table 1), but not in the first half of the tritonymph stage, the first and second half of the protonymph stage, or the first and second half of the larval stage (Table 1).

### 3.2 Experiment 2: Is size‐dependent male morph expression subject to allometric plasticity?

We reared 128 males to adulthood on filter disks soaked in a yeast concentration of 40 mg/ml (*n* = 35), 16.0 mg/ml (*n* = 21), 6.40 mg/ml (*n* = 12), 2.56 mg/ml (*n* = 37), and 1.02 mg/ml (*n* = 23). The interaction between QTIW and food quality was statistically significant (LRT: χ^2^ = 11.78, df = 4, *p* = .019), indicating that the effect of QTIW on the probability of fighter expression varied across food treatments. Upon visual inspection of the data, the significant interaction between QTIW and food quality was likely caused by two artifacts of limited sampling: (i) the effect of QTIW on fighter expression had vanished in 6.40 mg/ml. However, in this treatment we could sample only 12 males, four of which became scramblers and one expressing the intermorph phenotype; (ii) QTIW completely separated fighters (*n* = 27) from scramblers (*n* = 7) in 40.0 mg/ml. Therefore, we assumed the true interaction effect to be zero (constraining the slopes of the logistic regression curves to the same value) and proceeded to test the main effects of QTIW and food quality.

**FIGURE 3:**
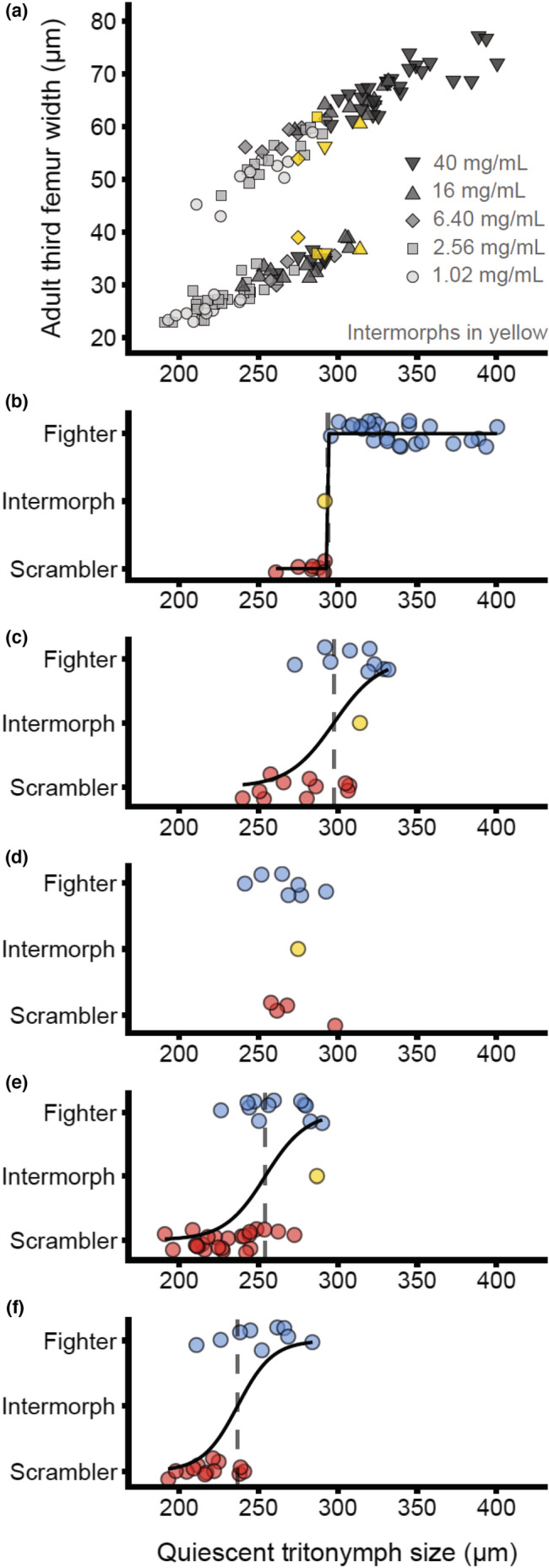
Condition‐dependent enlarged fighter leg development shows food‐dependent allometric plasticity. (a) The allometric relationship of adult male third femur width (left and right leg averaged) and body size at metamorphosis. Color and shape denote food quality (filter paper soaked in yeast solution; see legend). Intermorph males are shown in yellow; in this case, both third legs are shown. (b‐f) Male morph expression (blue: fighter, red: scrambler, yellow: intermorph) as a response to quiescent tritonymph body size, for males raised on a diet of 40.0 mg/ml yeast (b), 16.0 mg/ml yeast (c), 6.40 mg/ml yeast (d), 2.56 mg/ml yeast (e) and 1.02 mg/ml yeast (f). Solid black lines represent statistically significant (α = 0.05) logistic regression lines and denote the probability of fighter expression. Vertical dashed lines represent the quiescent tritonymph size threshold at which the probability of fighter expression is estimated to be 50%. A slight vertical jitter was added to panels (b‐f)

The probability of fighter expression given the effect of food type increased significantly with QTIW (LRT: χ^2^ = 75.83, df = 1, *p* < .001; Figure 3a–f). Food quality also had a highly significant effect on the probability of fighter expression given the effect of QTIW (LRT: χ^2^ = 44.49, df = 4, *p* < .001), showing a plastic body size threshold that scaled with food quality (Figure 3a–f).

## 4 DISCUSSION

We hypothesized that food‐dependent allometric plasticity underlies size‐dependent male morph expression in *R. robini*. If this is the case, the allometry of adult third leg width and body size at the developmental stage when male morph expression is cued should change depending on perceived food quality. We found that expression of the fighter phenotype is associated with body size and growth rate during the second half of the final juvenile stage before metamorphosis, but not earlier during ontogeny. This suggests that male morph expression is cued immediately before or during metamorphosis, in the quiescent tritonymph developmental stage. We then tested whether the allometry of quiescent tritonymph body size and the width of the adult third legs shift with food quality. We found that the allometry did shift toward lower body sizes with a deterioration in food quality, confirming allometric plasticity in *R. robini* male weaponry.

The pattern of food‐dependent allometric plasticity can emerge from different developmental mechanisms. First, it is possible that male tritonymphs weigh and integrate multiple cues from the nutritional environment, to assess future mating chances and adaptively adjust development (Emlen, 1997). Hypothetically, nutrition and growth inform not only on a male's own future physical strength, but also on the strength of potential rivals, provided that these rivals experience the same nutritional circumstances. If adult dispersal is limited and local variation in food quality is high, a male's developmental decision to prepare for an aggressive mating tactic could then depend not just on its own body size, but also on its body size relative to the expected average, favoring adaptive allometric plasticity (Emlen, 1997).

A second explanation for the observed allometric plasticity is that males use only a single cue for fighter expression, which is not body size, but instead *correlates* with body size. If the association of the “true cue” and body size depends on food quality, allometric plasticity will emerge. This could happen, for example, if male morph expression is cued by an individual's resource budget during metamorphosis (Smallegange et al., 2019). Larger individuals will have larger resource budgets on average, but resource budgets are not expected to scale linearly with body size, because the energetic expenses associated with growth and metamorphosis also increase with size. Therefore, allometric plasticity could be a symptom of budget‐dependent development of enlarged and expensive fighter legs. The intermorph males are interesting in this respect. Intermorph phenotypes are clearly maladaptive as a single fighter leg cannot functionally pinch and pierce opponents. Presumably, in these individuals, the developmental switch leading to fighter leg development has been triggered incompletely or in only one side of their bodies, possibly because these individuals had a resource budget close to the critical threshold for fighter leg development.

Finally, apparent allometric plasticity could emerge if male morph expression and relative body size are both pleiotropically influenced by genes of large effect. If that is the case, allometries are effectively genetically determined, while nutrition simply shifts the genetically determined allometries with average body size. Male morph expression in *R. robini* is certainly heritable (Radwan, 1995, 2003; Skrzynecka & Radwan, 2016), and fighter expression has been shown to be associated with genetic diversity and potentially coadaptive gene complexes (Stewart et al., 2019) and the upregulation of genes involved in general metabolism (Joag et al., 2016). However, to our knowledge, there is currently no evidence suggesting that the link between male morph expression and growth rate is caused by pleiotropy, rather than developmental plasticity of leg development in response to growth, size, the resource budget, or food quality. Pleiotropic genetic control of growth and male morph expression seems improbable given the results of our first experiment. Nonetheless, it is likely that the proportion of growth variation explained by genetic effects is larger when the nutritional environment is stable over time (as in our experiment 2) than when the nutritional environment is strongly fluctuating (as in our experiment 1). Likewise, male morph expression may be largely genetically influenced in unfluctuating nutritional environments, and more strongly affected by nutrition in fluctuating nutritional environments. More work is needed to exclude the possibility that apparent allometric plasticity in fact reflects a genetically controlled allometry, driven by genes of large effect, pleiotropically affecting both growth and male morph expression. However, to our knowledge, such a genetically controlled allometry would be a previously unrecorded phenomenon in arthropods.

Allometric plasticity is hypothesized to be an important first step in the evolutionary diversification of allometric scaling relationships (Casasa & Moczek, 2019), and has been recorded in a few species with conspicuous sexual trait allometries. In the dung beetles *Onthophagus acuminatus* and *O. taurus*, the allometry of male horns and adult body size plastically shifts with food quality and average body size (Emlen, 1997; Moczek, 2002), so that the allometry tracks a shift in the body size distribution much like we describe for *R. robini*. These examples of allometric plasticity have been hypothesized to reflect adaptation to variation in the expected competitive environment, reflected by seasonally varying food (e.g., dung quality) (Emlen, 1997). Alternatively, they may reflect a developmental side‐effect due to additional (but food‐dependent) growth after secondary sexually selected traits have been cued by body size (Moczek, 2002). The opposite pattern of food‐dependent allometric plasticity has also been recorded, where allometric plasticity does not track shifts in the body size distribution, but rather amplifies condition‐dependence. For example, in the stalk‐eyed fly *Diasemopsis aethiopica* and the armed beetle *Gnatocerus cornutus*, the allometric scaling of sexual trait size is lowered in individuals raised on poor‐quality compared to individuals raised on good‐ quality food (Knell et al., 1999; Okada & Miyatake, 2010). Allometries can also change in response to seasonal change: in the stag beetle *Lucanus cervus*, the allometry of male mandibles and adult body size changes over the course of summer, so that males pupating later in the year develop smaller mandibles for their body size than males pupating early in the year (Hardersen et al., 2011). In this case, allometric plasticity is hypothesized to be adaptive due to time constraints on development in combination with allocation trade‐offs: the mating season is short, so late‐developing males should accelerate maturation at the expense of energetic investment in weaponry (Hardersen et al., 2011). Yet another form of allometric plasticity occurs in the mite species *Rhizoglyphus echinopus* and *Sancassania berlesei*, which exhibit the same size‐dependent fighter‐scrambler polyphenism as the closely related *R. robini*. In these species, but curiously not in *R. robini* (Radwan, 1995), fighter expression in large males is suppressed by pheromones from dense colonies, so that the allometry collapses into complete scrambler expression in dense colonies (Radwan, 1993, 2001). This response has been demonstrated to be adaptive in *S. berlesei*: in large groups, the fighter phenotype no longer yields higher mating success than the scrambler phenotype even for large males (Michalczyk et al., 2018), and instead becomes a liability (Radwan et al., 2000; Radwan & Bogacz, 2000).

The different forms of allometric plasticity showcase the developmental flexibility associated with allometric scaling, but also highlight that these apparently similar patterns may have evolved under different selection pressures. Therefore, to understand how allometric plasticity evolves, it is necessary to study these selection pressures in detail—that is, does the allometric scaling relationship of male sexual traits respond adaptively to different environmental cues for the expected future competitive environment? However, the evolution of allometric plasticity should be understood not just in terms of the selection pressures that might favor it, but also in terms of how allometric variation emerges from development. We may not even expect that allometries are food‐independent, if we factor in the likely possibility that the energetic expenses of enlarged sexual trait production, relative to the total resource budget, depend on body size and growth rate. In fact, if we take such a mechanistic approach to understand the ecology and evolution of allometric plasticity it means we must move away from quantitative genetic approaches like the environmental threshold (ET) model (Hazel et al., 1990, 2004) and its latent version (Buoro et al., 2012). The ET model takes conditionality as an environmentally cued liability exceeding a genetically determined threshold value. It illustrates well how conditionality is compatible with statistical evolutionary theory and is a valuable tool when predicting evolutionary shifts in conditionality (e.g., Smallegange & Deere, 2014; Tomkins et al., 2011). Yet it should not be interpreted mechanistically, for example, by treating body size as an environmental cue and “sensitivity to body size” as a genetically determined threshold, because the roles of “cue,” “liability,” and “threshold” may map poorly on developmental reality, and all are affected by genetic and environmental effects.

Our observation of food‐dependent allometric plasticity in *R. robini* adds to the limited number of recorded cases of allometric plasticity in male arthropod secondary sexual traits. Allometric plasticity is hypothesized to facilitate rapid local adaptation, adaptive divergence, and speciation, if allometric plasticity occurs in the same direction as evolutionary change of trait allometries, and if plastic shifts in allometries coincide with release of cryptic genetic variation (Casasa & Moczek, 2019; Noble et al., 2019). To understand this potentially important evolutionary process, we should start looking for allometric plasticity in different species with conspicuous allometries in variable environments, and investigate whether the direction of allometric plasticity (Emlen, 1997; Hardersen et al., 2011; Knell et al., 1999; Moczek, 2002; Okada & Miyatake, 2010) generally coincides with evolutionary shifts in the allometry (Moczek et al., 2002; Moczek & Nijhout, 2003; Smallegange & Deere, 2014; Tomkins et al., 2011). Only then can we start to understand how development produces allometric variation, how or whether selection favors particular plastic responses of the scaling relationship, and eventually whether allometric plasticity contributes to evolutionary diversification.

## AUTHOR CONTRIBUTIONS

**Flor T. Rhebergen:** Conceptualization (lead); data curation (lead); formal analysis (lead); investigation (lead); methodology (lead); visualization (lead); writing – original draft (lead); writing – review and editing (equal). **Kathryn A. Stewart:** Conceptualization (supporting); investigation (supporting); supervision (supporting); writing – original draft (supporting); writing – review and editing (equal). **Isabel M. Smallegange:** Conceptualization (supporting); investigation (supporting); resources (lead); supervision (lead); writing – original draft (supporting); writing – review and editing (equal).

## CONFLICT OF INTEREST

We declare no conflict of interest.

## Supporting information


Data S1

## Data Availability

Data are available at Figshare: https://doi.org/10.6084/m9.figshare.20179841.v1
